# Case Report: Long-term remission of malignant pleural and peritoneal effusion in a case of advanced lung adenocarcinoma treated with combined crizotinib and anlotinib therapy

**DOI:** 10.3389/fonc.2023.1191522

**Published:** 2023-08-08

**Authors:** Jin Tian, Lin Long, Jianhua Zang, Peng Liu, Lili Zhao, Hongtao Zhang, Jun Xiao

**Affiliations:** ^1^ Oncology Center I Department, Qingdao Hiser Hospital Affiliated of Qingdao University (Qingdao Traditional Chinese Medicine Hospital), Qingdao, Shandong, China; ^2^ Department of Radiotherapy, Qingdao Hiser Hospital Affiliated of Qingdao University (Qingdao Traditional Chinese Medicine Hospital), Qingdao, Shandong, China; ^3^ Department of Pharmacy, Qingdao Hospital, University of Health and Rehabilitation Sciences (Qingdao Municipal Hospital), Qingdao, Shandong, China

**Keywords:** malignant pleural effusion, malignant peritoneal effusion, crizotinib, Anlotinib, non-small cell lung cancer

## Abstract

Malignant pleural and peritoneal effusion are common clinical manifestations in advanced malignant tumors, associated with a poor prognosis. This article presents a case of advanced lung adenocarcinoma with ROS1 rearrangement, characterized by persistent malignant pleural and peritoneal effusion. The patient received combined therapy of Crizotinib and Anlotinib, resulting in a significant reduction and even disappearance of the malignant effusion. Exploratory use of this treatment approach improved the patient’s quality of life and holds potential for extending overall survival. However, given the single case report nature, the efficacy of this regimen in treating advanced ROS1-rearranged lung adenocarcinoma should be considered as a supplementary strategy, warranting further validation through multicenter clinical data.

## 1 Introduction

Non-small cell lung cancer (NSCLC) ranks among the most prevalent and fatal malignancies worldwide, accounting for 80%-90% of cancer cases (1). Malignant pleural and peritoneal effusion can result from local progression or distant metastasis of malignant tumors. Studies have consistently shown that patients with concurrent malignant pleural and peritoneal effusion have a significantly worse prognosis, with a median survival ranging from 3 to 6 months (2). Moreover, malignant effusion adversely impacts respiratory, circulatory, and digestive functions, diminishing patients’ quality of life and compromising the effectiveness of anticancer treatments. Comprehensive management strategies for malignant pleural and peritoneal effusion encompass thoracentesis, closed chest drainage, and pleural catheter placement. Therefore, devising appropriate treatment approaches for effectively managing malignant effusion is pivotal to enhance cancer patients’ quality of life and improve treatment outcomes.

In recent years, significant progress has been made in the field of targeted cancer therapy, substantially prolonging the survival of patients with specific genetic mutations. ROS1 gene rearrangements occur in approximately 1-2% of NSCLC patients and represent validated therapeutic driver mutations (3). Crizotinib, a multitargeted tyrosine kinase inhibitor (TKI) targeting MET/ALK/ROS1, has demonstrated notable efficacy in treating NSCLC with ROS1 rearrangements. Anlotinib, a novel multitargeted TKI developed in China, effectively inhibits kinases such as vascular endothelial growth factor receptor (VEGFR), platelet-derived growth factor receptor, fibroblast growth factor receptor, and c-Kit, exerting its actions on tumor angiogenesis and growth inhibition (4). Although the combination therapy of Crizotinib and Anlotinib has garnered research attention for small cell lung cancer (SCLC), its clinical application remains limited. Crizotinib targets the anaplastic lymphoma kinase (ALK) gene rearrangement, while Anlotinib is a multitargeted agent that inhibits multiple growth factor receptors and angiogenesis-related pathways. *In vitro* experiments and animal models have suggested a potential synergistic effect of combining Crizotinib and Anlotinib in SCLC treatment. This therapeutic approach holds promise by simultaneously targeting multiple key pathways implicated in tumor growth and angiogenesis, thereby enhancing treatment efficacy.

Following an extensive review of databases such as PubMed, Web of Science, and CNKI, we identified limited literature on the use of Crizotinib and Anlotinib for treating malignant effusion in lung adenocarcinoma in China. Here, we report a case of ROS1-rearranged lung adenocarcinoma with concurrent malignant pleural and peritoneal effusion. The patient achieved complete remission of effusion and long-term relief following maintenance therapy with Crizotinib and Anlotinib.

## 2 Case report

The patient, a 58-year-old male, was admitted to the Department of Thoracic Surgery at Qingdao Municipal Hospital on June 8, 2017. Upon admission, the patient presented with symptoms of cough, chest tightness, and persistent shortness of breath lasting for over a month. There were no apparent triggering factors in the patient’s work environment, and there was no family history of cancer or genetic disorders. The primary symptom reported throughout the course of the illness was respiratory distress. The initial treatment approach involved thoracentesis for pleural effusion drainage, which provided relief for the patient’s chest tightness and respiratory symptoms.

A chest enhanced CT scan revealed multiple enlarged lymph nodes in the right pulmonary hilum and mediastinum, multiple nodules in the right pleura, as well as pleural effusion. However, no obvious occupying lesions were seen in both lungs. Further examination of the pleural fluid cytology confirmed the presence of cancer cells. Histological images showed tumor cells can be seen under the microscope, with significant heterogeneity. Some cells are arranged in solid masses or small cords, some are visible as glandular lumen formation, and some are arranged in tubular or adenoid structures. Immunohistochemical results showed the tumor characteristics as follows: Ki-67 (5%+), TTF-1 (+), NapsinA (+), CK7 (+), CR (-), MC (-), Vim (-), EMA (+), CK (+), and CK5/6 (-). Physical examination: no thoracic deformity, bilateral respiratory motion symmetry, turbid sounds on right lung percussion, disappearance of breath sounds in the middle and lower lungs, clear sounds on left lung percussion, no dry and moist rales were heard.

According to the American Joint Committee on Cancer (AJCC) staging system, the patient’s tumor size cannot be measured, the tumor metastasis is pleura, and pleural effusion is present, and the lymph node invasion is ipsilateral to the hilar and mediastinum, taking into account the patient’s symptoms, imaging findings, pleural fluid characteristics, immunohistochemistry, and staging guidelines, the patient was diagnosed with advanced-stage malignant tumor in the right lung (adenocarcinoma, stage IV A). The patient’s treatment journey has spanned over 5 years since June 8, 2017.

**Figure 1:**
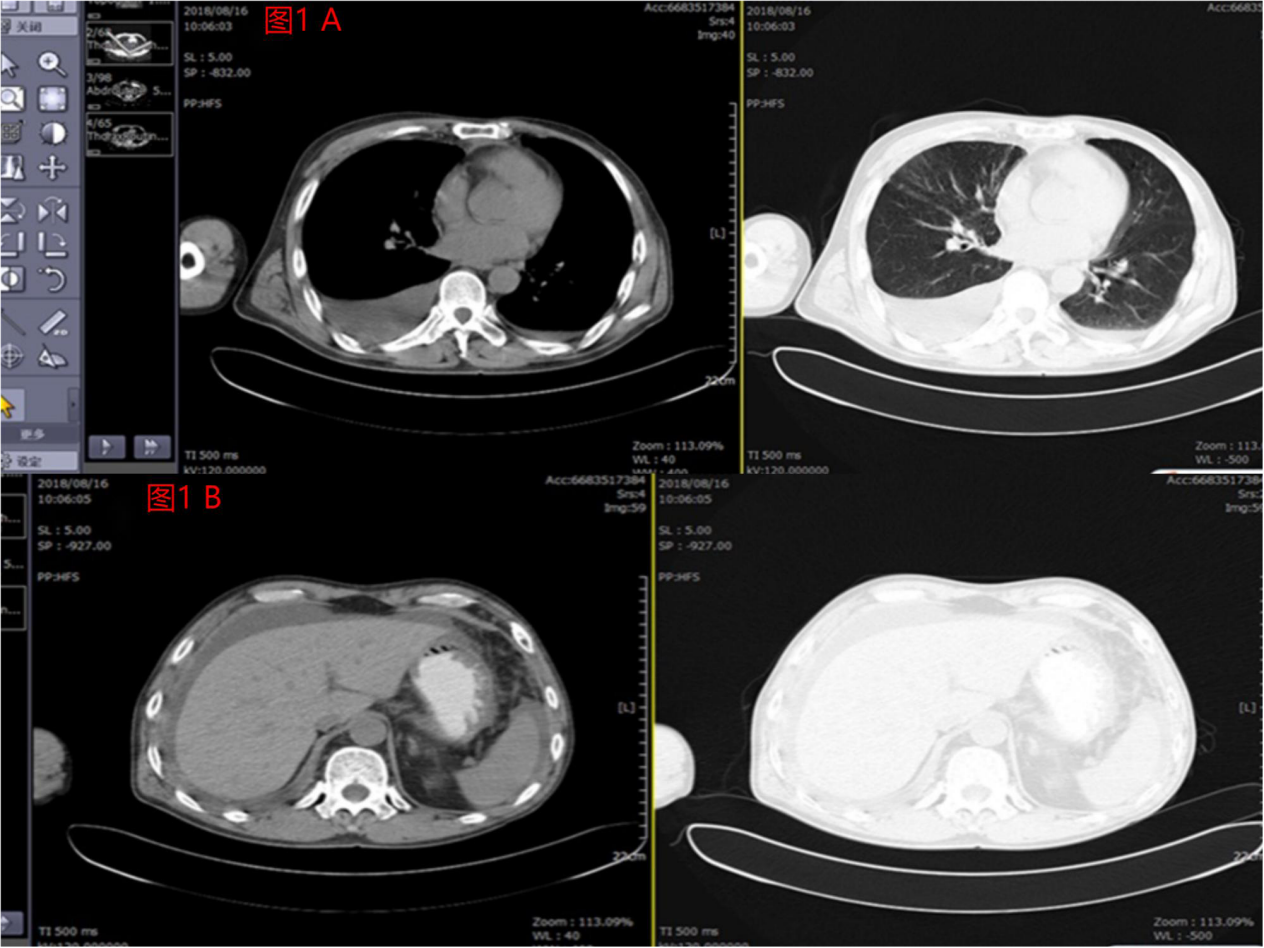
**(A)** Pleural effusion was shown by imaging findings before the patient underwent crizotinib combined with anlotinib treatment in August 2018. **(B)** Significantly large perihepatic effusion was shown by imaging findings before the patient underwent crizotinib combined with anlotinib treatment in August 2018.

The main treatment modality for the patient has been chemotherapy, consisting of multiple cycles. The first chemotherapy cycle was initiated on June 17, 2017, involving pemetrexed 800mg on day 1 and nedaplatin 60mg on day 1. On July 12, 2017, the patient received intrathoracic injections of Endo 45mg and Ganoderma lucidum polysaccharide 4mg. Subsequently, from July to August 2017, the patient underwent 2-3 cycles of chemotherapy with pemetrexed 900mg on day 1 and carboplatin 700mg on day 1. Genetic testing revealed no alterations in EGFR/ALK/ROS1.Between September 2017 and January 2018, the patient underwent 4-7 cycles of intravenous chemotherapy, comprising pemetrexed 900mg on day 1, cisplatin 50mg on day 1, and bevacizumab 600mg. Additionally, intrathoracic injections of cisplatin 80mg and bevacizumab 200mg were administered. After completing 7 cycles of chemotherapy, the patient experienced significant improvement in ascites symptoms and a reduction in dyspnea. On March 6, 2018, the patient received Endo 30mg on days 1-7 and pemetrexed 800mg on day 4, as per the medical instructions. However, the patient did not adhere to the prescribed treatment. Subsequent evaluation revealed an increase in tumor markers, and PET-CT indicated disease progression. Therefore, an additional 2 cycles of chemotherapy were administered using the original regimen: Endo 30mg on days 1-7 and pemetrexed 800mg on day 3. Following the completion of this treatment cycle, the patient was readmitted due to abdominal distension and dyspnea. Subsequent CT scans revealed the presence of malignant pleural and peritoneal effusion (**Figure 1**; **Supplemental Figure**).

**Figure 2:**
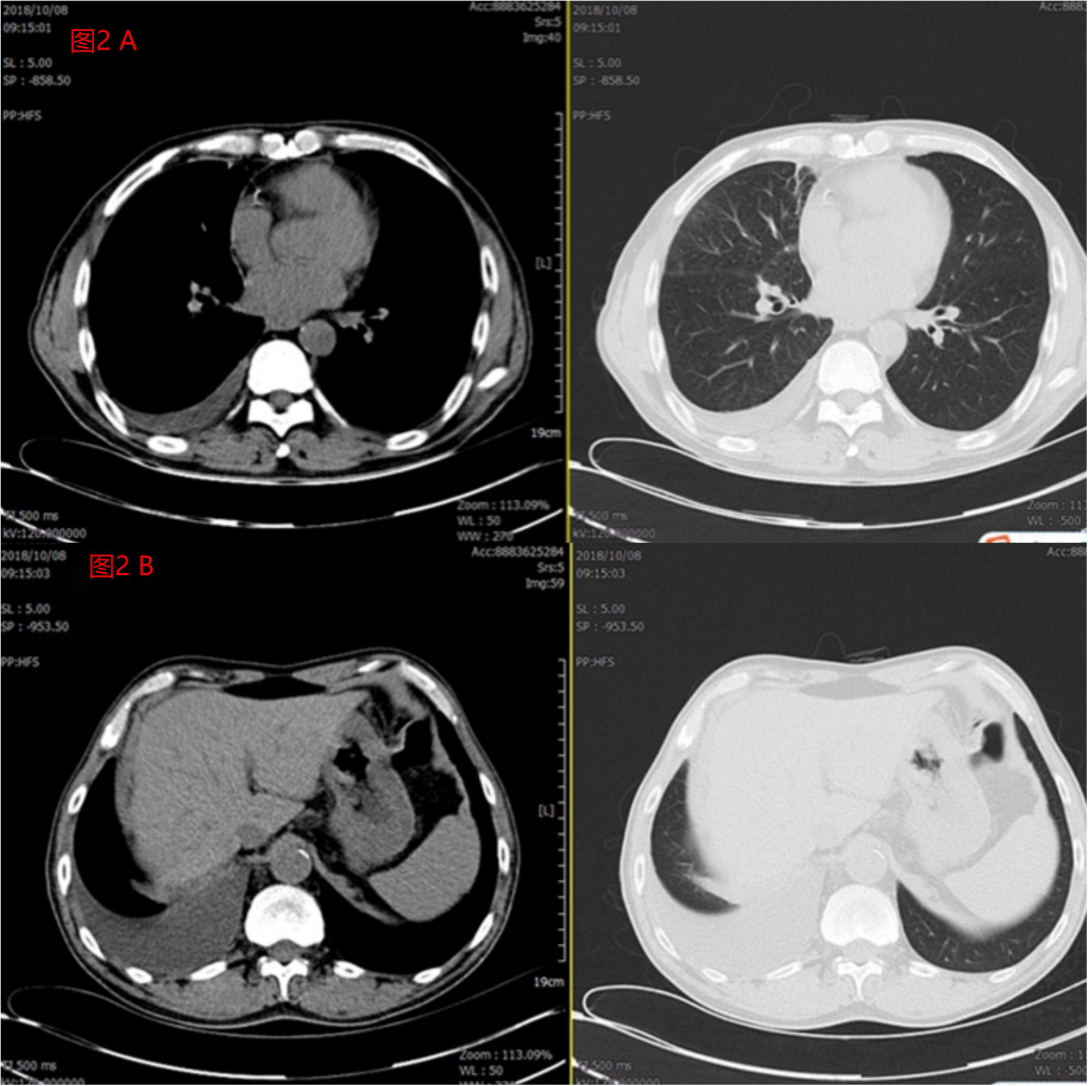
**(A)** The patient’s pleural effusion was significantly reduced after 1 month of treatment compared to pre-treatment. **(B)** The patient’s peritoneal effusion was significantly reduced after 1 month of treatment compared to pre-treatment.

On August 21, 2018, the patient underwent palliative chemotherapy with cisplatin 40mg on day 1 and 50mg on days 2-3. Due to recurrent pleural effusion and the presence of peritoneal effusion, intraperitoneal infusion of Endo 105mg on day 1 and day 5 was performed on August 24, 2018. The follow-up CT scan showed tumor infiltration of the intestinal tract. Attempted high-throughput sequencing of blood samples revealed a ROS1 rearrangement. Starting from September 5, 2018, the patient initiated targeted therapy with crizotinib 250mg twice daily in combination with anlotinib 10mg once daily. The treatment resulted in significant symptom relief, and the malignant pleural and peritoneal effusion decreased compared to the CT results on August 21, 2018 (**Figure 2**).

Continuing with the targeted therapy regimen of crizotinib 250mg twice daily and anlotinib 10mg once daily (with a two-week administration of anlotinib followed by a one-week break), the patient’s chest and lung signs were evaluated. Subsequent follow-up visits indicated the absence of dyspnea, and imaging examinations of the thoracic and abdominal regions showed no significant malignant infiltrations for over 4 years. The patient developed grade 2 hypertension during the medication period, which was controlled by oral antihypertensive drugs. As of March 2023, the patient’s disease has progressed, and palliative treatment has been administered.

## 3 Diagnosis/follow-up and outcomes

Diagnosis: 1. Pulmonary malignancy stage IV A 2. Malignant pleural effusion 3. Malignant peritoneal effusion 4. Metastatic colonic malignancy

Follow-up and outcomes: The patient is currently undergoing palliative and nutritional support treatment in the Department of Oncology, Qingdao Municipal Hospital for various reasons.

## 4 Discussion

Lung cancer is a highly prevalent and deadly malignancy in both China and globally, with non-small cell lung cancer (NSCLC) accounting for approximately 80% of cases. About 5% of NSCLC tumors involve rearrangements in the ALK gene located on chromosome 2 (5). In 2007, Soda (6) identified the EML4-ALK fusion gene in NSCLC, where the most common ALK rearrangement involves fusion of the 5’ end of the EML4 gene with the 3’ end of the ALK gene, resulting in the formation of the fusion oncogene EML4-ALK (7). This fusion protein triggers dimerization of the intracellular kinase domains of ALK, activating downstream oncogenic signaling and leading to disease progression and poor prognosis (8).

The formation of malignant pleural and peritoneal effusions is closely associated with vascular endothelial growth factor (VEGF). Tumor proliferation and metastasis are often accompanied by increased vascular density, and VEGF promotes tumor neovascularization by secreting various pro-angiogenic factors, including VEGFA (9). The elevated vascularity of tumor blood vessels contributes to the development of malignant pleural effusion (10). Moreover, VEGF can bind to receptors on mesothelial epithelial cells, increasing their permeability and facilitating fluid reflux, thus disrupting the dynamic equilibrium between fluid production and absorption.

The patient in this case is a middle-aged male with advanced lung adenocarcinoma. Despite undergoing seven cycles of platinum-based chemotherapy, his disease continued to progress, and the control of pleural and peritoneal effusions was suboptimal. Initial genetic testing yielded negative results, but given the worsening condition, a second round of genetic testing revealed a ROS1 rearrangement. The patient received combined treatment with Anlotinib and Crizotinib to control pleural and peritoneal effusions, resulting in a significant reduction in fluid accumulation after one month of treatment. The patient achieved stable long-term relief, with no serious adverse reactions during the course of drug administration.

The combination of Anlotinib and Crizotinib offers several advantages and limitations. As multitarget tyrosine kinase inhibitors, both drugs can simultaneously inhibit multiple key targets such as VEGFR, EGFR, PDGFR, ROS1, and ALK. This multitarget inhibition mechanism may lead to a more comprehensive anti-tumor effect and improved treatment outcomes, particularly in tumors with multiple aberrant signaling pathways. Additionally, combining Anlotinib with Crizotinib may help overcome resistance observed with Crizotinib monotherapy by suppressing chemokine-mediated angiogenesis and enhancing the overall anti-tumor efficacy (11). However, it is important to note that the combined use of Anlotinib and Crizotinib may increase the risk of adverse reactions, as both drugs have their own side effect profiles including hypertension, hand-foot syndrome, fatigue, and gastrointestinal discomfort. Furthermore, the efficacy of the combination treatment may vary depending on individual tumor characteristics and genetic variations. While preliminary studies have reported favorable results with Anlotinib combined with Crizotinib in lung cancer treatment, larger-scale clinical trials and long-term follow-up data are still lacking to establish the effectiveness and safety of this combination therapy. Therefore, further research is warranted to validate its therapeutic efficacy and determine the optimal treatment strategy.

The patient received multiple cycles of intravenous Endo and Bevacizumab, as well as local injections of Bevacizumab and Endo into the abdominal and pleural cavities, but the outcomes were unsatisfactory. Based on the clinician’s experience, the addition of Anlotinib to Crizotinib led to unexpected treatment efficacy for the patient. The combination of Anlotinib and Crizotinib represents a promising approach for ALK-TKI-resistant patients.

In conclusion, Combined treatment with Crizotinib and Anlotinib may demonstrate effectiveness in late-stage ROS1-rearranged lung adenocarcinoma with concurrent malignant pleural and peritoneal effusions, significantly prolonging survival and improving quality of life, as shown in the present case. After an initial negative genetic testing result, targeted therapy should not be permanently abandoned, and a second round of genetic testing should be considered. The clinical practice presented in this case may provide novel insights into the treatment of malignant effusions, although further research is needed to elucidate the treatment mechanism and confirm its efficacy.

## Data availability statement

The original contributions presented in the study are included in the article/**Supplementary Material**. Further inquiries can be directed to the corresponding authors.

## Ethics statement

The patient did not have any objection to the treatment plan, fully understood his condition, and expressed happiness and satisfaction with receiving Anrotinib check crizotinib treatment and obtaining long-term remission, and will strictly follow medical advice, actively treat and live optimistically in the future. Written informed consent was obtained from the participant/patient(s) for the publication of this case report.

## Author contributions

JT and LL are in charge of data acquisition and data analysis. JZ/PL and LZ are in charge of literature review, clinical data collection and validation. JT made a contribution to manuscript editing. HZ and JX are in charge of manuscript review. All authors contributed to the article and approved the submitted version.
