# Supplementary figures and images for: Case Report: Long-term remission of malignant pleural and peritoneal effusion in a case of advanced lung adenocarcinoma treated with combined crizotinib and anlotinib therapy
Source: Front Oncol. 2023 Aug 8;13:1191522. doi: 10.3389/fonc.2023.1191522 (PMC10444195; doi:10.3389/fonc.2023.1191522)

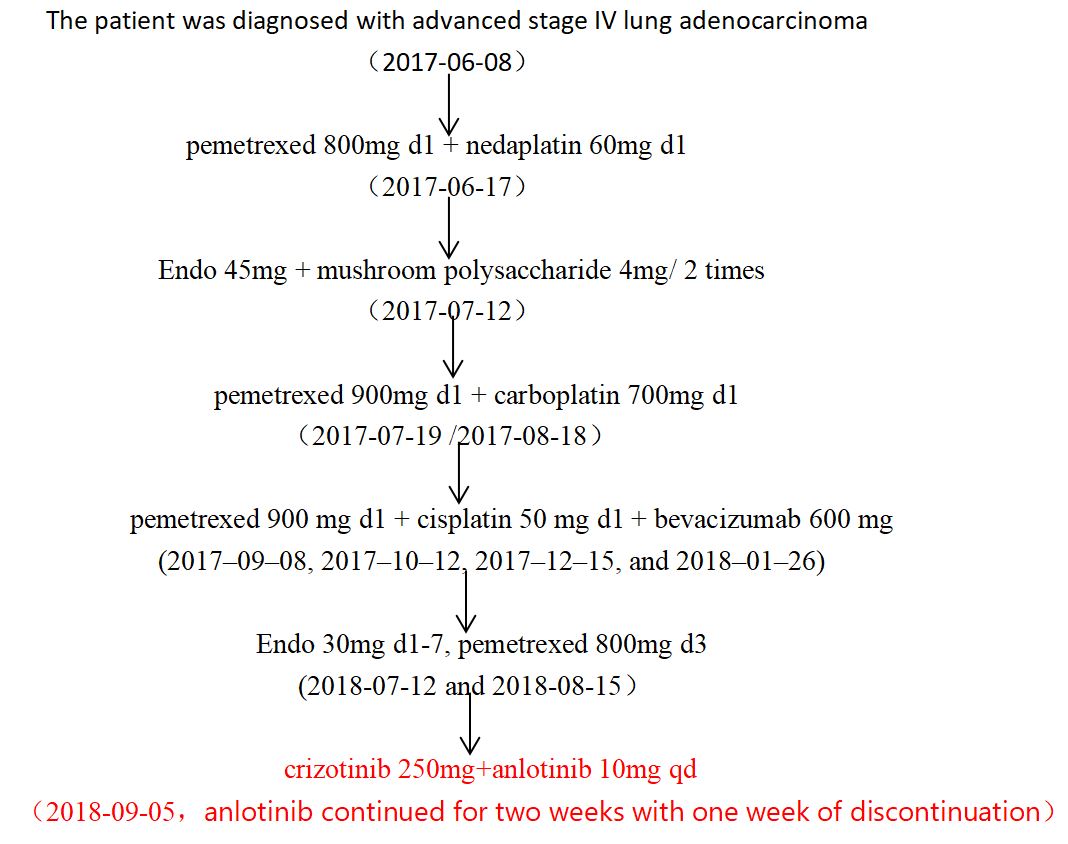

Supplement: Supplementary file 1 [file Image_1.jpeg]
